# *dab2* is required for the scavenging function of lymphatic endothelial cells in the zebrafish meninges

**DOI:** 10.1038/s41598-024-76590-9

**Published:** 2024-11-14

**Authors:** Katharina Uphoff, Irina Suárez, Andreas van Impel, Stefan Schulte-Merker

**Affiliations:** Institute for Cardiovascular Organogenesis and Regeneration, Faculty of Medicine, University of Münster, Röntgenstraße 16, 48149 Münster, Germany

**Keywords:** Angiogenesis, Lymphangiogenesis, Lymphatic system, Mutation

## Abstract

To date it is only partially understood how the brain is cleared of waste products resulting from its high metabolic activity, although this process has important implications for the development and progression of neurodegenerative diseases. Lymphatic vessels play a central role in maintaining fluid and tissue homeostasis, and the recent description of meningeal lymphatic vessels within the dura mater of mice, human and zebrafish has raised considerable interest in unraveling the function of these vessels. In zebrafish, brain lymphatic endothelial cells (BLECs) constitute an additional meningeal lymphatic endothelial cell population. These highly endocytically active cells contribute to the clearance of the brain, but the molecular basis of this scavenging activity is only poorly understood. Here, we report on the characterization of zebrafish *disabled 2* (*dab2*) mutants. Embryos lacking maternally provided *dab2* show defective venous sprouting from the caudal vein plexus at 26hpf. Furthermore, we show that the cargo-specific adaptor protein is specifically expressed in BLECs, and that BLECs are significantly impeded in their capacity to internalize specific substrates injected into the cerebrospinal fluid upon loss of zygotic *dab2*. Our work therefore identifies Dab2 as an important member of the molecular machinery mediating the scavenging function of BLECs in the meninges.

**Supplementary Information:**

The online version contains supplementary material available at 10.1038/s41598-024-76590-9.

## Introduction

The lymphatic system constitutes a unidirectional, blind-ended vascular network in vertebrates, that is in charge of maintaining fluid homeostasis, transporting immune cells and absorbing dietary fats in the intestine^1^. To maintain fluid homeostasis, the lymphatic network takes up interstitial fluid from tissues and directs it back to the venous blood circulation. The interstitial fluid contains, among other constituents, toxic molecules and cellular debris, and hence lymphatic vessels also contribute to waste removal from tissues and organs. The lymphatic system serves vital functions and is thus present in almost all vascularized organs, with a few noticeable exceptions, most notably the brain parenchyma^2^. How the brain is cleared from waste generated by its high metabolic activity has long been an active line of research and alternative theories to traditional lymphatic waste removal have been formulated^3,4^. Recently, the topic has risen lots of interest again due to the discovery of lymphatic vessels in the meninges of mice, humans and zebrafish^5–7^. To which extent these meningeal lymphatic vessels contribute to the clearance of the brain parenchyma to maintain homeostasis, and how they work together with other proposed clearance mechanisms is still under debate. Another level of complexity has been added with the description of a particular population of lymphatic endothelial cells (LECs) with scavenging function in the zebrafish meninges, which take up substances from the cerebrospinal fluid intracellularly into lysosomal vesicles and contribute, together with microglia, to tissue homeostasis in the central nervous system^8,9^. These cells are known alternatively as brain lymphatic endothelial cells (BLECs), mural lymphatic endothelial cells (muLECs), and Fluorescent Granular Perithelial cells (FGPs)^9–11^. BLECs transdifferentiate bilaterally at 56 h post fertilization (hpf) from the venous endothelium of the choroidal vascular plexus and migrate following the mesencephalic vein. At 5 days post fertilization (dpf) they arrange into characteristic symmetric loops that cover the optic tectum (TeO) and they also populate the midbrain-hindbrain boundary and the ventral side of the brain. During further development, this population expands over the fore-, mid-, and hindbrain covering the whole surface of the brain after three weeks of age^9–11^. Throughout all these stages, BLECs do not form vessels as one would expect from LECs, but rather form a network of single cells. Interestingly, BLECs are always located at the level of the meninges, but have been reported to enter the brain parenchyma temporarily to resolve complications resulting from certain pathological conditions^12^.

As for the zebrafish trunk lymphatic vasculature, the development of BLECs depends on Vascular endothelial growth factor C (Vegfc) and its receptor Vascular endothelial growth factor receptor 3 (Vegfr3)^9,10^. Moreover, BLECs express well known lymphatic markers such as *vegfr3*,* prospero homeobox protein 1 (prox1)*, and *hyaluronic acid receptor 1 (lyve1)*, and their formation is unaffected by depletion of the hematopoietic lineage. A recent study has reported the existence of cells within mouse and human leptomeninges with very similar structural characteristics and gene expression profiles as zebrafish BLECs, although further investigation is needed to prove that they indeed represent the same cell type^13^.

Scavenger endothelial cells (SECs) are important for the innate and adaptive immune system and are known as the major clearance site of macromolecules, colloidal waste and viral particles from the blood circulation^14,15^. In zebrafish embryos, SECs are located in large veins like the posterior cardinal vein and the common cardinal vein. Recently, we have shown that BLECs perform similar scavenger functions in the leptomeninges as SECs do in the blood vasculature^8^. BLECs possess a very high endocytic activity^9–11^ making them even more efficient in the uptake of small molecules from the cerebrospinal fluid than microglia^8^. The precise mechanism of how BLECs accomplish their scavenging function still needs to be fully elucidated.

Clathrin-mediated endocytosis is a central mechanism to internalize cargo smaller than 500 nm^16^. During receptor mediated endocytosis, extracellular molecules are bound to (cargo-)receptors within the plasma membrane and via association with specific adaptor proteins that recruit clathrin molecules to so-called clathrin-coated pits. These are internalized and in turn will give rise to endocytic vesicles. A prominent example of a cargo-specific adaptor protein is Disabled homolog 2 (DAB2), which has sequence and domain similarities with other cargo-specific adaptor proteins^17^. One of these conserved domains is the Phospho-Tyrosine Binding domain (PTB) which is located at the N-terminus and which recognizes NPXY motifs present in a variety of transmembrane proteins, including the LDL receptor, integrins, and the EGF receptor. Because of its ability to bind ligand/receptor complexes, DAB2 has a role in modulating signalling cascades. In fact, it has previously been shown that DAB2 binds VEGFR3 upon activation by VEGF-C and that, in *Dab2* knock-down endothelial cells, VEGFR3 internalization and downstream activation of the small GTPase Rac1 and the protein kinases ERK1/2 is impaired^18^. In mice models, loss of *Dab2* causes embryonic lethality before gastrulation, due to DAB2 being required for the internalization of critical cell adhesion molecules^19^. At later stages, once embryonic development has been completed, DAB2 appears to not be essential anymore, but different studies have shown that it is involved in the uptake of cholesterol and the re-uptake of LRP2/Megalin in the kidney^20^. However, the phenotype of those mice is very mild, possibly because of redundancy with similar adaptor proteins. In zebrafish, Dab2 was shown to be involved in molecule internalization in the pronephric duct^21^ and in the internalization of Bmp2 in venous endothelial cells^22^.

Because of the link between DAB2 and the VEGF-C/VEGFR3 signalling pathway and the role of DAB2 in clathrin-mediated endocytosis, the present study aimed to elucidate the involvement of Dab2 in the clearance activity of zebrafish BLECs. Here, we show that zygotic *dab2* is not essential for embryo survival, venous sprouting from the caudal plexus, or development of the lymphatic vasculature. However, lack of maternal *dab2* affects venous sprouting from the caudal vein plexus at 26hpf. Moreover, we demonstrate that zygotic *dab2* mutants, despite showing elevated numbers of BLECs, have significant defects in macromolecule internalization from the cerebrospinal fluid suggesting an important role of Dab2 for the scavenging activity of this cell population.

## Results

### ***dab2*** is expressed in BLECs

**Fig. 1:**
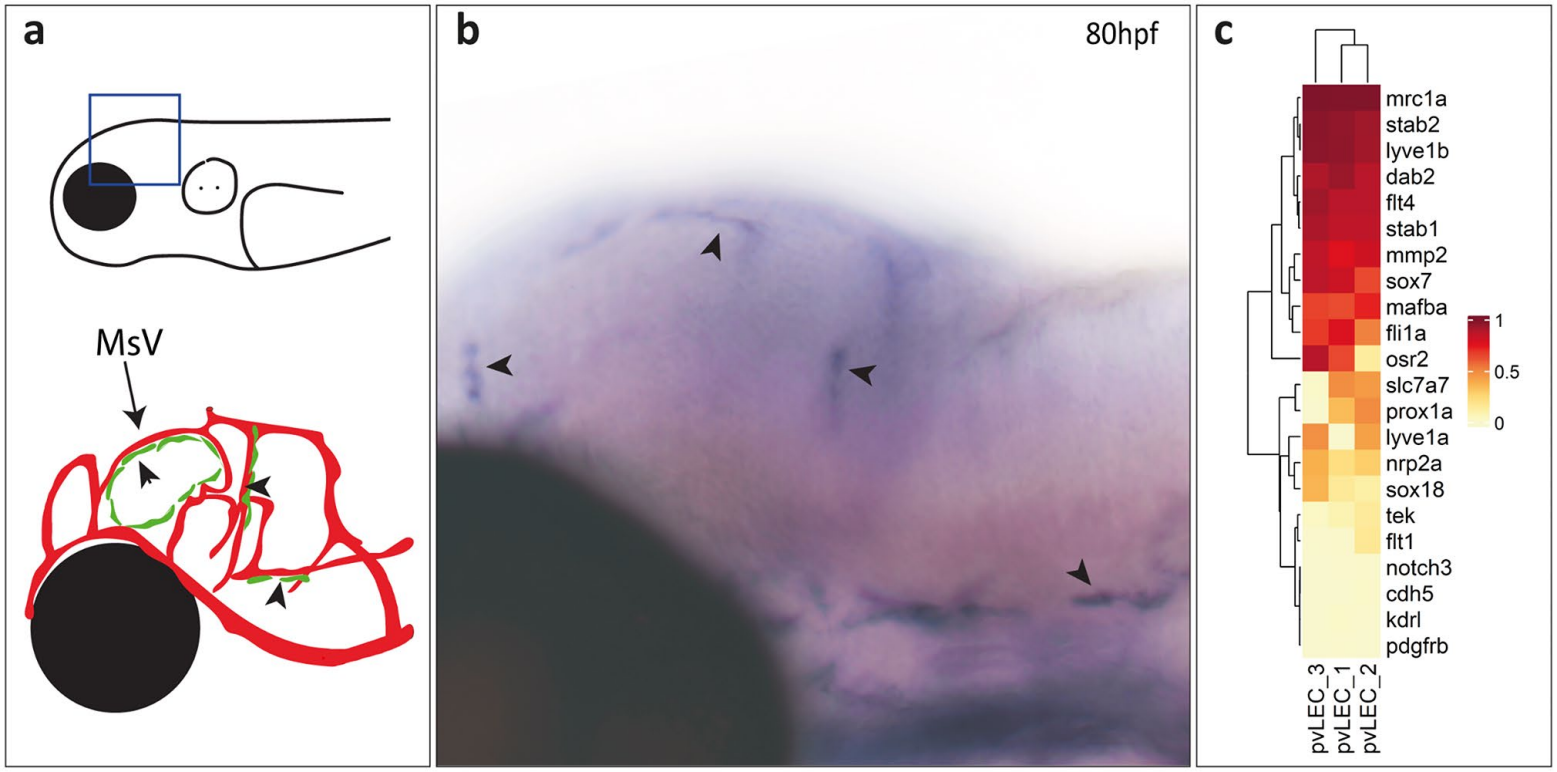
*dab2* is expressed in BLECs. (**a**) Schematic representations of a 4dpf zebrafish embryo head from a lateral view. In the top drawing, a blue square marks the region shown magnified in the bottom drawing and in (b). The bottom drawing depicts BLECs (green, arrowheads) that are arranged in loops at the dorsal side of the TeO, at the midbrain-hindbrain boundary and on the ventral side of the hindbrain. Major blood vessels are illustrated in red. (**b**) Lateral view of an in situ hybridization against *dab2* transcripts, highlighting *dab2* expression within BLECs in *casper*-/- embryos at 80hpf (arrowheads). (**c**) Heat-map representing relative gene expression levels within BLECs (YuGene data published by Bower et al.^10^) for known lymphatic endothelial markers (*lyve1b*, *prox1a*, *flt4*, *mafba*, *stabilin1*, *stabilin2*, *lyve1a*, *nrp2a*, *mmp2*, *mrc1a*, *ors2*, *slc7a7*), the pan-endothelial marker *fli1a* and several genes not expressed by lymphatic endothelial cells (*flt1*, *notch3*, *cdh5*, *kdrl*, *pdgfrß*). Note the high *dab2* expression levels in BLECs. The colour scale indicates relative expression from 1 (highest) to 0 (lowest). BLEC, brain lymphatic endothelial cell; dpf, days post fertilization; hpf, hours post fertilization; MsV, mesencephalic vein; TeO, optic tectum.

Given that Dab2 has a reported function during endocytosis in endothelial cells, we wondered whether it is also expressed by BLECs, a population of scavenger endothelial cells that takes up cargo very efficiently via receptor-mediated endocytosis^9^. In situ hybridization (ISH) performed on zebrafish embryos at 80hpf showed *dab2* transcripts in individual cells located dorsally to the optic tectum and forming bilateral loops, at the midbrain-hindbrain boundary, and on the ventral side of the hindbrain, all positions which are characteristic for the localization of BLECs at this stage^8,9^ (Fig. 1a and b). To verify that these cells are indeed BLECs, we performed an ISH against *dab2* followed by an immunofluorescent staining of *mrc1a:mCit* positive cells (Supplementary Fig. 1) and found that all *mrc1a:mCit*-positive BLECs do express *dab2* transcripts. Analysis of previously published RNA-sequencing data derived from FACS-isolated BLECs from 12-month-old zebrafish^10^ confirmed that *dab2* is highly expressed in BLECs even at adult stages (Fig. 1c).

### ***dab2***^***mu410***^ represents an RNA null allele

Having demonstrated that *dab2* is expressed in BLECs, we intended to generate a *dab2* mutant line to study the effects of loss of Dab2 activity on BLEC function in zebrafish.

**Fig. 2:**
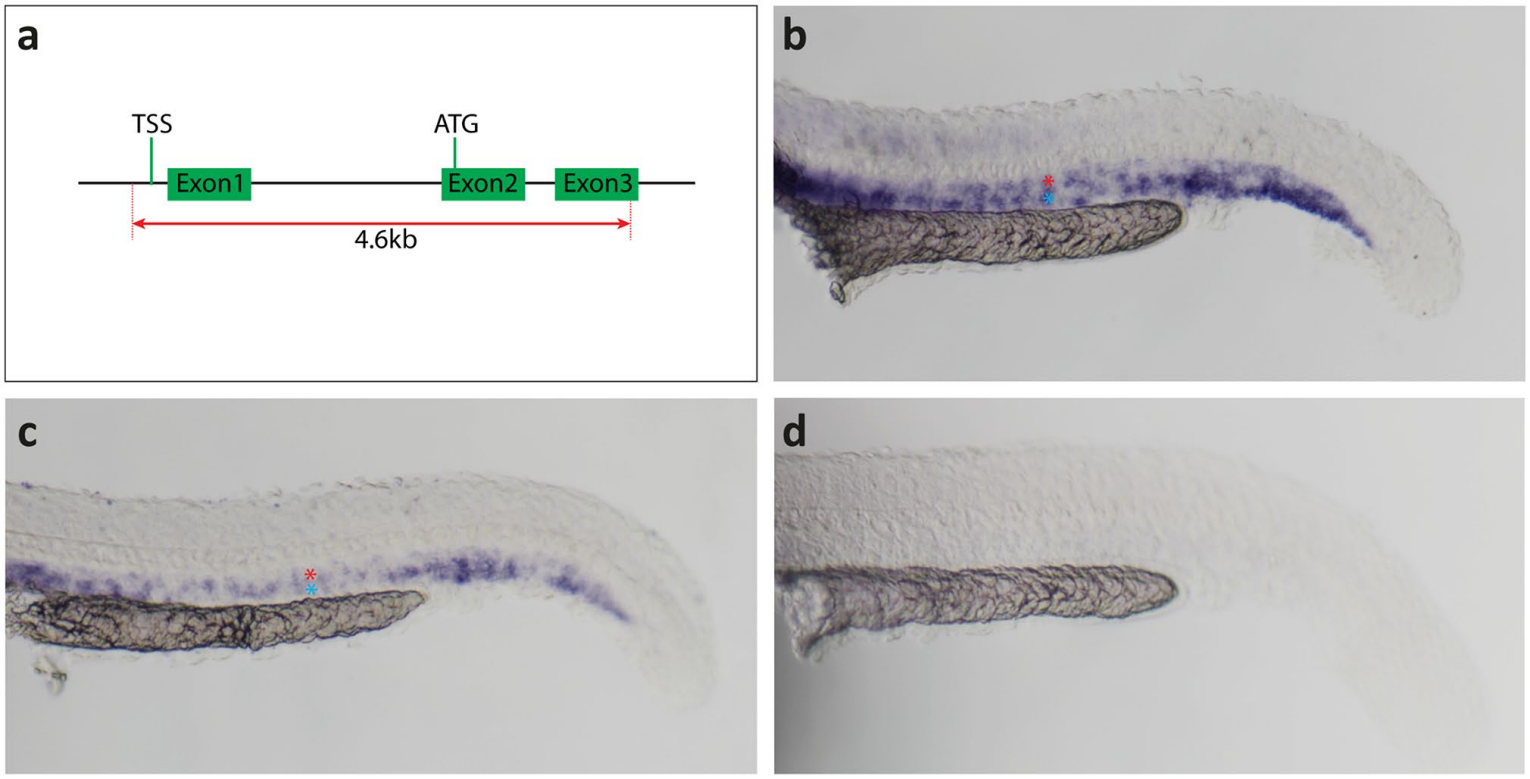
Generation of the *dab2*^*mu410*^ allele results in a mRNA null situation. (**a**) Scheme for the generation of the *dab2* promoter knock-out. Two gRNAs (red dotted lines) were designed to remove the TSS and first translated ATG, resulting in a deletion of a 4.6 kb genomic fragment including the *dab2* promoter. (**b-c**) Images of ISH stainings with a *dab2* RNA antisense probe. At 24hpf, *dab2* is expressed in the DA (red asterisk) and the PCV (blue asterisk) of wild type (b) and heterozygous (c) embryos, whereas no staining is detectable in homozygous mutants (d). ATG, first translated ATG; DA, dorsal aorta; hpf, hours post fertilization; ISH, in situ hybridization; PCV, posterior cardinal vein; TSS, transcription start site.

Since RNA fragments generated by nonsense-mediated decay of a mutant mRNA can trigger the activation of compensatory genes thereby masking the phenotype caused by the mutation of a gene^23^, we generated a *dab2* promoter deletion allele (*dab2*^*mu410*^*)* using CRISPR/Cas9 with two gRNAs flanking a 4.6 kb region containing the transcription start site (TSS) and the first translated ATG of *dab2* (Fig. 2a). In situ hybridization experiments employing 24hpf embryos revealed that *dab2* is expressed in the dorsal aorta and the posterior cardinal vein of wild type and heterozygous embryos (Fig. 2b and c), in agreement with previously published data^22^. No staining was observed in homozygous *dab2*^*mu410*^ mutant embryos (Fig. 2d), indicating that the mutants do not express *dab2* mRNA and thus suggesting that *dab2*^*mu410*^represents an RNA-null allele.

### Caudal vein defects in embryos lacking maternally provided ***dab2*** mRNA

**Fig. 3:**
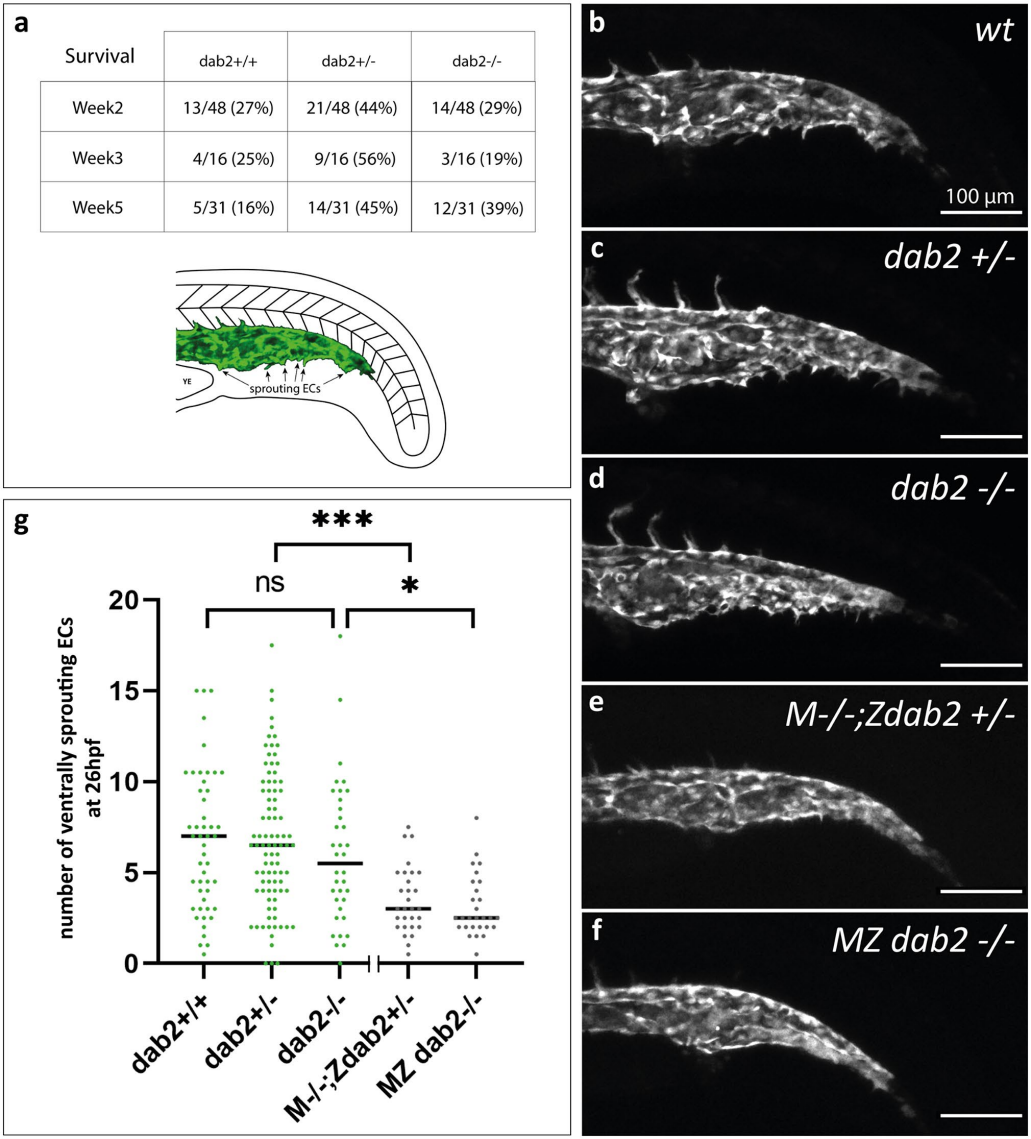
Formation of the caudal vein plexus is severely reduced in embryos that lack maternally provided *dab2* mRNA. (**a**) Number of wild type, heterozygous and homozygous mutant fish after genotyping the progeny from a *dab2*+/- incross at week 2, 3 or 5 of development. At all time points, there is a high percentage of homozygous mutants. Schematic representation of the caudal vein area in the zebrafish trunk at 26hpf. In green, the caudal vein with ventrally sprouting ECs is represented. (**b-f**) Maximum projection of *flt4:mCitrine* positive zebrafish embryos at 26hpf, lateral view. Wild type embryos (**b**), *dab2+/-* (**c**), and *dab2-/-* (**d**) embryos develop sprouting ECs that bud off ventrally from the caudal vein. In comparison, M*-/-*;Z*dab2+/-* (**e**) and MZ *dab2-/-* (**f**) embryos develop fewer sprouts. (**g**) Quantification of the number of ventrally sprouting ECs reveals that the number is significantly lower in maternal mutant embryos (Kruskal Wallis, Mann Whitney U-test; M*-/-*;Z*dab2+/-* vs. *dab2 +/-p* = 0.000195 and MZ *dab2-/-* vs. *dab2 -/-p* = 0.014). The graph shows the median and all the embryos quantified (dots). hpf, hours post fertilization; MZ maternal-zygotic; YE, yolk extension.

In order to characterize this novel *dab2* mutant allele, we first checked for previously published phenotypes that were detected upon knock-down of *dab2* transcripts. Utilizing an ATG blocking morpholino, Kim et al.^22^ described defects in the formation of the caudal vein plexus, a structure that is generated by ventrally budding endothelial cells from the caudal vein at 25hpf^24^. We therefore imaged the offspring of heterozygous *dab2*^*mu410*^ fish at 26hpf and quantified the number of ventrally sprouting ECs in the caudal vein (Fig. 3).

In zygotic *dab2* mutants (*dab2-/-*) ventrally sprouting ECs within the caudal vein were visible and did not show overt differences to wild type (*dab2+/+*) and heterozygous (*dab2+/-*) siblings (Fig. 3b-d). Subsequent quantification of positions with ventrally sprouting endothelial cells within the caudal vein area confirmed this initial observation as the number of sprouts was not significantly reduced in mutant embryos (Fig. 3g: *dab2 +/+* Median = 7, *n* = 49 vs. *dab2-/-* Median = 5.5, *n* = 36; [Mann-Whitney U test *p* = 1]). Since this is in stark contrast to the defects seen after morpholino injection, we thought to exclude the possibility that maternally provided *dab2* transcripts would rescue the caudal vein plexus formation in zygotic *dab2* situations, utilizing homozygous *dab2* mutant females. In contrast to systemic loss of *Dab2* in mice, which leads to embryonic lethality between E6.0-6.5 ^19^, we found that the survival rates of the offspring of heterozygous *dab2*^*mu410*^ in-crosses were as expected from a Mendelian inheritance at two, three and five weeks of age, suggesting that homozygous *dab2* mutants are fully viable and fertile in zebrafish (Fig. 3a). Making use of such homozygous *dab2* mutant females we analysed crosses to heterozygous *dab2* males. In the resulting embryos lacking all maternally provided *dab2* transcripts, a defective caudal vein plexus formation was observed, independent of the zygotic genotype of the embryos (Fig. 3e-f). The zygotic heterozygous offspring of this *dab2* mutant female will hereafter be referred to as *M-/-;Zdab2+/-* and the zygotic homozygous offspring as *MZ dab2-/-*. Quantifications revealed fewer positions with actively sprouting ECs in *M-/-;Zdab2+/-* embryos compared to *dab2+/-* embryos. Likewise, numbers were also significantly reduced in *MZ dab2-/-* embryos when compared to zygotic *dab2-/-* embryos, (Fig. 3g: *M-/-;Zdab2*+/- Median = 3, *n* = 29 vs. *dab2 +/-* Median = 6.5, *n* = 89; [Mann-Whitney U test *p* = 0.000195]; *MZ dab2-/-* Median = 2.5, *n* = 28 vs. *dab2 -/-* Median = 5.5, *n* = 36; [Mann-Whitney U test *p* = 0.014] and *dab2 +/+* Median = 7, *n* = 49 vs. *MZ dab2-/-* Median = 2.5, *n* = 28 [Mann-Whitney U test *p* < 0.0001]). It is important to note, that zygotic and maternal-zygotic embryos can never be siblings from the same cross, hence the comparison of both groups was carried out with embryos that were derived from two different, but simultaneous matings. Taken together, our analysis shows that after loss of maternally provided *dab2*, venous sprouting from the caudal vein is strongly reduced, a finding in line with the data generated by Kim and colleagues (2012) and furthermore, indicating that maternally provided *dab2* can rescue vascular defects in zygotic *dab2* mutants.

### ***dab2***^***mu410***^ mutants possess elevated numbers of BLECs

**Fig. 4:**
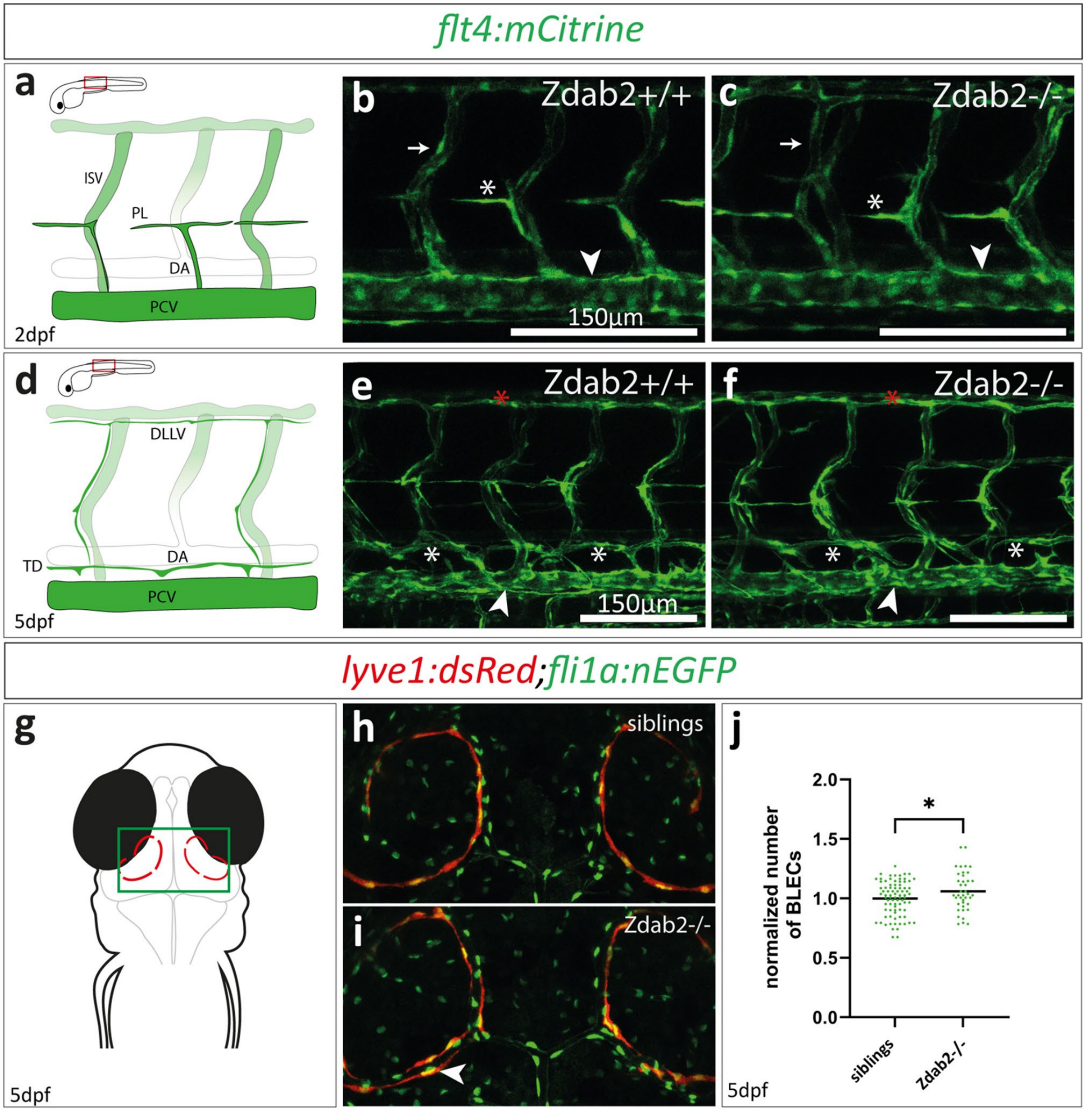
Zygotic *dab2* mutants do not show lymphatic defects but exhibit elevated numbers of BLECs. (**a**) Schematic representation of a wild type trunk vasculature at 2dpf indicating the zoomed in area in b and c (red rectangle). (**b**,** c**) Maximum projection of confocal images of wild type (b) and homozygous *dab2* mutants (c) with PL cells (white asterisk) positioned at the HM at 2dpf. The PCV (arrowhead) and ISVs (arrow) are marked as reference structures. (**d**) Schematic representation of the wild type trunk vasculature at 5dpf. (**e**,** f**) Maximum projection of confocal images of *flt4:mCitrine*-positive embryos at 5dpf. Wild type fish (**e**) and homozygous *dab2* mutants (**f**) have formed the TD (white asterisks) dorsally to the PCV (arrowhead) as well as the DLLV (red asterisks). (**g**) Schematic drawing of a zebrafish head at 5dpf (dorsal view) with BLECs depicted in red, highlighting the area shown in (**h**) and (**i**). (**h**,**i**) Maximum projection of confocal images taken at 5dpf. Note the *lyve1:dsRed* signal in BLECs (red) and the *fli1a*:*nEGFP* expression marking all endothelial nuclei. While BLECs are visible in both, siblings (**h**) and homozygous *dab2* mutants (**i**), *dab2* mutants occasionally show a second row of BLECs (arrowheads). (**j**) Quantification of BLECs reveals a significant increase in BLEC numbers in *dab2* mutants (Unpaired t test; siblings vs. *dab2* -/- *p* = 0.0418). BLEC, brain lymphatic endothelial cell; DLLV, dorsal longitudinal lymphatic vessel; dpf, days post fertilization; HM, horizontal myoseptum; ISV, intersegmental vessel; PCV, posterior cardinal vein; PL, parachordal lymphangioblast; TD, thoracic duct; DA, dorsal aorta.

Since *dab2* expression becomes more restricted to the venous domain after 32hpf^22^ and since *dab2* has been reported to affect the activation of the major lymphangiogenic pathway^18^, i.e. the Vegfc/Vegfr3 signalling axis, we decided to investigate the formation of lymphatic structures in zygotic *dab2* mutant embryos. Formation of the lymphatic vasculature in the zebrafish trunk begins with the migration of endothelial cells that sprout from the cardinal vein during a process called lympho-venous (or secondary) sprouting (Fig. 4a). We found that in *dab2*^*mu410*^ mutant embryos venous endothelial cells migrated to the horizontal myoseptum, where they aligned temporarily as lymphatic precursor cells termed parachordal lymphangioblasts (PLs), exactly as in wild type embryos (Fig. 4b and c). The analysis of PL numbers in zygotic *dab2-/-* embryos at 48hpf did not reveal significant differences between wild type and mutant *dab2* embryos (Supplementary Fig. 2). Subsequently, PLs migrated dorsally and ventrally along arteries to form the dorsal longitudinal lymphatic vessel (DLLV), the thoracic duct (TD), as well as the intersegmental lymphatic vessels (Fig. 4d) in *dab2* mutant embryos, indistinguishable from the wild type situation by 5dpf (Fig. 4e and f). However, when analysing *M-/-;Zdab2+/-* and *MZdab2-/-* embryos we found a mild but significant reduction in PL numbers compared to the zygotic mutant and heterozygous situation (Supplementary Fig. 2), suggesting that the Vegfc/Vegfr3 dependent formation of these lymphatic structures is sensitive to Dab2 activity, which in zygotic mutants is covered by maternally provided *dab2* mRNA.

Finally, we assessed the consequences of loss of *dab2* on the development of the BLEC population (Fig. 4g). Similar to the other lymphatic structures analysed, we found that the sprouting and development of BLECs is not drastically affected in *dab2* mutants, as BLECs are visible in the characteristic loop structures dorsally to the optic tectum by 5dpf. However, we noticed that 35% of the zygotic *dab2* mutant embryos displayed a second row of BLECs, suggesting an increased number of these cells (Fig. 4h and i). Making use of a transgenic line with a nuclear marker (*fli: nEGFP)*, we subsequently quantified the number of BLECs, and found that the number was indeed significantly higher in *dab2* mutants compared to their siblings (Fig. 4j: *siblingsn* = 82 vs. *dab2 -/-n* = 40; data normalized to siblings [Unpaired t test *p* = 0.042]). Taken together, our analysis of zygotic *dab2* mutants revealed no overt defects in the major lymphatic vessels but a significant impact on the number of BLECs populating the leptomeninges.

### ***dab2***^***mu410***^ mutant BLECs exhibit defects in the uptake of substances from the cerebrospinal fluid

**Fig. 5:**
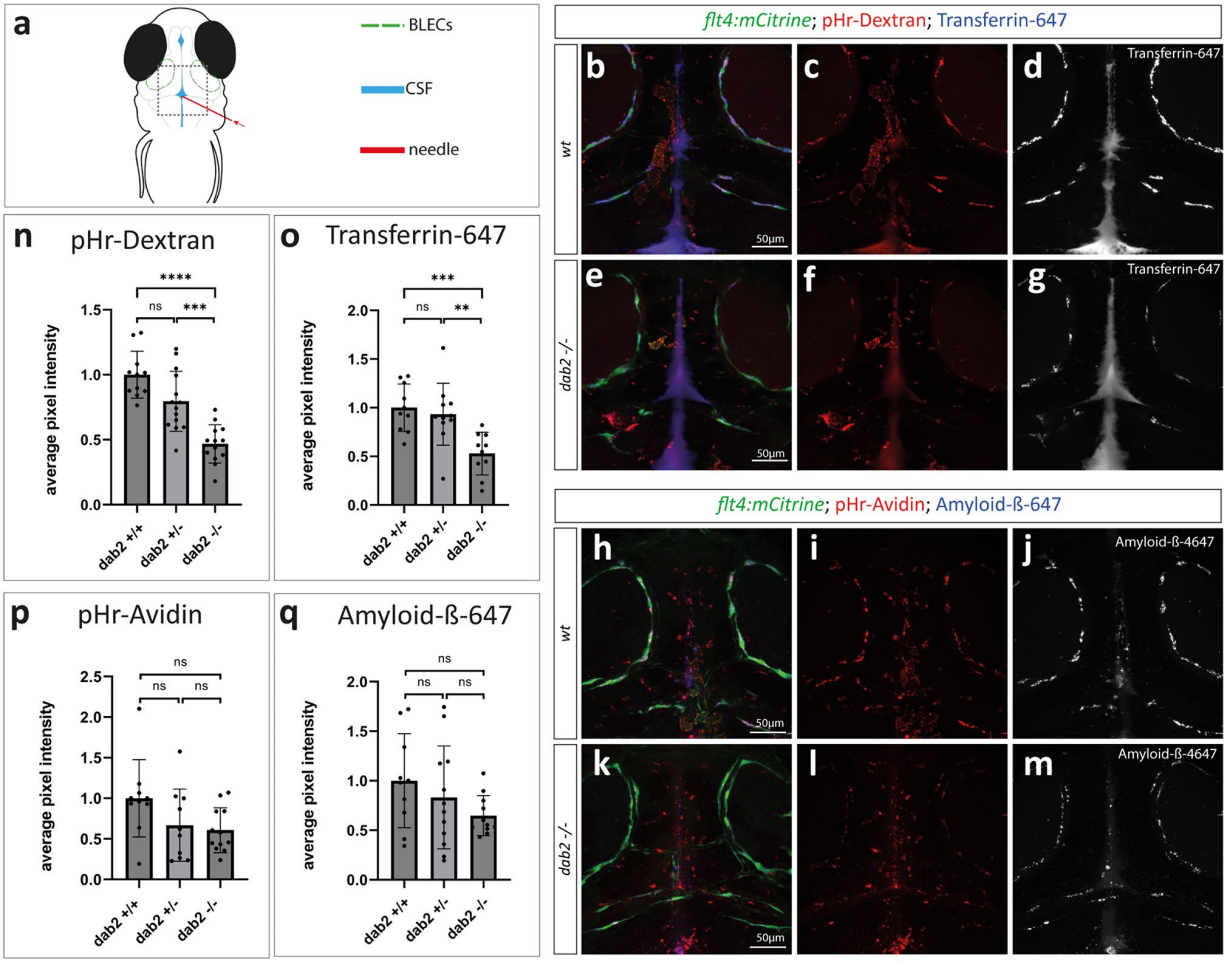
Internalization of different fluorescently labelled substances from the CSF is affected in BLECs devoid of Dab2. (**a**) Schematic representation of a 5dpf zebrafish embryo from a dorsal view indicating the injection site (red) into the CSF (blue). BLECs are represented in green. The dotted rectangle marks the region of imaging. (**b**,**e** and **h**,**k**) Maximum projection of a *flt4:mCitrine* positive wild type sibling (b-d, h-j) or *dab2-/-* (e-g, k-m) zebrafish embryo injected either with pHr-Dextran and Transferrin-647 (b-g) or with pHr-Avidin and Amyloid-ß-647 (h-m) at 5dpf. Images were taken 1hpi. For each substance, single channels of the respective composite pictures are shown separately. Note the higher signal intensity within BLECs in wild type (c, d, i, j) compared to *dab2-/-* embryos (f, g, l, m). **(n-q)** Quantification of pHr-Dextran (n) and Transferrin-647 (o) uptake in BLECs reveals significantly higher average pixel intensities in wild type compared to *dab2-/-* embryos (ANOVA, t-test; pHr-Dextran: *p* = 2e-7, Transferrin-647: *p* = 0.0008). The difference in average pixel intensities for Avidin (p) and Amyloid-ß-647 (q) between wild type and to *dab2-/-* embryos is not statistically significant (Kruskal Wallis, Mann Whitney U-test; pHr-Avidin: *p* = 0.09, Amyloid-ß-647: *p* = 0.296). Graphs show the mean ± SD of 2 (Amyloid-ß) to 3 (Dextran, Transferrin, Avidin) replicates and the individual data point for each embryo (dots). The average pixel intensities are normalized to wild type embryos. CSF, cerebrospinal fluid; dpf, days post fertilization; hpi, hours post injection; pHr, pH-rodo; SD, standard deviation.

Given the high expression levels of *dab2* in BLECs and the known function of Dab2 as an adaptor protein during clathrin-mediated endocytosis, we hypothesized that the loss of *dab2* might affect the ability of BLECs to internalize cargo from the brain parenchyma and cerebrospinal fluid, which in turn might trigger a compensatory response leading to the significant increase in BLEC numbers. To examine the endocytic capability of BLECs that lack Dab2, different fluorescently labelled substances were injected into the cerebrospinal fluid (Fig. 5a) of the offspring of *dab2*^*mu410*^ heterozygous zebrafish at 5dpf, which were subsequently imaged one hour after injection. Intracellular uptake of Dextran and Transferrin (Fig. 5b-g) and Avidin and Amyloid-β (Fig. 5h-m) by BLECs was monitored by co-injections of two substances of which one was coupled to pHrodo. Since endocytic vesicles gradually acidify on their way to the lysosomal compartment, the pH-sensitive pHrodo, which strongly fluoresces in acidic environments, served as a control for the successful internalization of the tested substances, in case both signals overlapped.

We noticed that all tested substances exhibited lower intensities within BLECs of *dab2*^*mu410*^ mutant embryos compared to their siblings one hour after injection, suggesting an overall lower uptake efficacy of BLECs after loss of functional Dab2 (Fig. 5e-g and k-m). To confirm this observation, the average pixel intensity (api) of each dye was quantified within BLECs (Fig. 5n-q). This analysis revealed a significant reduction in the uptake of Dextran and Transferrin in *dab2* mutants compared to their wild type siblings. For Avidin and Amyloid-β, only a mild reduction was noticeable, however not statistically significant (Fig. 5n: dextran *dab2+/+* 1 api vs. *dab2-/-* 0.468 api [ANOVA, unpaired t-test *p* = 2e-7]; Fig. 5o: Transferrin *dab2+/+* 1 api vs. *dab2-/-* 0.529 api [ ANOVA, unpaired t test *p* = 0.0008]; Fig. 5p: Avidin *dab2+/+* 1api vs. *dab2-/-* 0.607 api [Kruskal Wallis, Mann Whitney U-test *p* = 0.09]; and Fig. 5q: Amyloid-β *dab2+/+* 1 api vs. *dab2-/-* 0.648 [Kruskal Wallis, Mann Whitney U-test *p* = 0.296]). Taken together these results show that loss of Dab2 impairs the scavenging function of BLECs, suggesting an involvement of the adaptor protein in this process.

## Discussion

The monomeric clathrin-associated adaptor protein DAB2 is involved in processes such as endocytosis of extracellular material and was shown to modulate cell signalling events via mediating the internalization of ligand/receptor complexes, including VEGFR3, a central player during lymphangiogenesis^18^. Since BLECs represent a scavenger endothelial cell population of lymphatic origin that exhibits a high endocytic activity, we wondered whether Dab2 is required in this context.

Our initial analysis showed active expression of *dab2* mRNA in BLECs at 80hpf. This is in line with RNA-sequencing data of zebrafish embryos^25^ that also indicated expression of *dab2* in BLECs during embryonic stages. Additionally, our analysis of RNA-sequencing data of BLECs generated by Bower et al. (2017) demonstrated that *dab2* is also highly expressed in BLECs of adult zebrafish, suggesting that *dab2* expression in BLECs persists from embryonic to adult stages. To test for Dab2 function, we generated a zebrafish *dab2* promoter deletion mutant via CRISPR/Cas9 mediated genome editing to circumvented possible complication as expression and subsequent degradation of mutant mRNA can lead to transcriptional compensation^26^. Embryos harbouring the deletion were negative in in situ hybridization experiments, demonstrating an RNA null situation. Nevertheless, *dab2* promoter knock-out zebrafish mutants are viable and survive until adulthood at expected Mendelian ratios. Adult *dab2* mutant fish are fertile, do not show any overt phenotype, and are indistinguishable from their siblings upon visual inspection, suggesting only non-essential functions for *dab2* during zebrafish development. This is in contrast to the situation after systemic loss of *Dab2* in mice which causes early embryonic lethality at E6.0-6.5, as embryos fail to gastrulate after implantation^27^. *DAB2* is expressed in the peripheral endoderm layer of E4.5-5.5 mouse embryos and is critical for the development of extra-embryonic endoderm^19^. While in wild type mice the *Dab2*-expressing primitive endoderm cells form a polarized epithelium and differentiate into visceral and parietal endoderm cells^19^, in *Dab2* deficient mice the primitive endoderm cells are present but fail to position on the surface to form a primitive visceral endoderm epithelium^19^. Interestingly, the presence of DAB2 in extra-embryonic tissue can rescue *Dab2-/-* mouse embryos efficiently, showing that *Dab2* is required in visceral endoderm but is dispensable in the embryo proper^27^. The fact that zebrafish have extra-uterine development and are not dependent on nutrient transport from mother to offspring after birth might therefore explain why zebrafish *dab2* mutants develop and survive to adulthood. This difference makes it possible to study further functions of Dab2 during vessel development in zebrafish, which in mice is only possible by utilizing vessel-specific *CreERT2*-transgenic lines crossed into a line with a loxP-flanked *Dab2* gene background^18^.

It had previously been reported by Kim et al. (2012) that, while the overall vascular structure of *dab2* morphants is normal, defects are evident during caudal vein plexus formation, as shown by a reduced number of ventrally sprouting ECs. The analysis of zygotic *dab2* mutants, however, did not show a significant reduction in the number of ventrally sprouting ECs compared to wild type and heterozygous *dab2* embryos. Since Kim et al. (2012) had shown that *dab2* is already present before the maternal-to-zygotic transition and thus maternally provided, the quantification of the ventrally sprouting ECs was also performed in embryos that lack maternally provided mRNA. In this case, a significant reduction in the number of ventrally sprouting ECs is evident that is comparable to the *dab2* morphant phenotype. The ATG blocking morpholino used by Kim et al. (2012) binds at the ATG start site of the *dab2* mRNA transcript thereby blocking its translation and hence affecting both, maternally and zygotically provided *dab2* mRNA. Taken together, these results demonstrate that the maternally provided, but not the zygotic expression of *dab2* mRNA is essential and sufficient for normal caudal vein plexus formation. Thus, in zebrafish, Dab2 is required for early embryonic development, but this requirement is camouflaged in zygotic mutants due to the maternal contribution of *dab2*.

As mentioned before, the adaptor protein DAB2 is involved in clathrin-mediated endocytosis^20^, and indeed, a connection between DAB2 and the internalization of the vascular receptor tyrosine kinases VEGFR3 and VEGFR2 has already been demonstrated by Nakayama et al. (2013). In their study, utilizing the murine retina as a model, they reported lower VEGFR2 immunosignal at the sprouting front of growing retinal vasculature in comparison to quiescent proximal vessels, reflecting differences in turnover rate (degradation and new synthesis) of VEGFR2. High receptor turnover and signalling rates can be important for leading migrating cells that need to respond quickly to signals in their environment^18^. It has been suggested that differences in the regulation of VEGFR endocytosis through ephrin-B2, DAB2, PAR-3 and aPKC help to assign specific tasks to endothelial cells. In mice, vessel-specific inhibition of *Dab2* reduces the endothelial uptake of labelled VEGF-A after intra-ocular injections and knockdown of DAB2 or PAR-3 in cell culture leads to a decreased uptake of VEGF and reduced ERK1/2 and RAC1 activation. These results led to the suggestion of an endocytic trafficking complex comprised of DAB2, PAR3, and VEGFR2 or VEGFR3^18^.

Since lymphangiogenesis is dependent on Vegfc/Vegfr3 signalling^28^, lymphatic structures were assessed in the newly generated *dab2* promoter deletion mutants. Lymphangiogenesis in zygotic *dab2* mutants was not affected in the trunk area. Furthermore, BLECs bud from the choroidal vascular plexus in absence of Dab2. However, quantification of the number of BLECs demonstrated that cell numbers were elevated in *dab2* mutants. This might be a sign for a compensatory mechanism in response to the impaired uptake capacity of the BLECs in *dab2* mutants to maintain homeostasis in the brain parenchyma by expanding the number of BLECs in the leptomeninges. We therefore analysed whether this monomeric clathrin-associated adaptor protein is involved in the scavanging function of BLECs by injecting different fluorescently labelled substances into the cerebrospinal fluid of 5dpf zebrafish embryos. This has previously been demonstrated to provide an efficient read-out for the uptake of exogenous substances by BLECs^8,9^. Whereas the uptake of Amyloid-β and Avidin was not significantly affected, there was a significant impairment in the internalization of Dextran and Transferrin injected in *dab2* promoter deletion mutants.

Given that the uptake of cargo molecules is not completely blocked in the absence of *dab2*, it is likely that other adaptor proteins can compensate for the loss of Dab2. Such a case has been reported for the internalization of the low-density lipoprotein receptor (LDLR) in mice models^29^. In this study, only slight effects in the internalization of LDLR in Dab2 conditional mice mutants were reported, whereas the simultaneous deletion of Dab2 and the adaptor protein Arh completely blocked LDLR internalization and resulted in mice with hypercholesterolemia. Interestingly, this is in agreement with the observations made by Maurer and Cooper, who described the absence of AHR in the visceral endoderm of mice, which could explain the complete blocking of Transferrin internalization in the absence of DAB2 in this tissue^30^. It therefore remains to be determined whether Arh constitutes an alternative adaptor protein during clathrin-mediated endocytosis in BLECs, or whether other related molecules can compensate for the loss of Dab2. However, our results suggest that BLECs make use of a very complex and redundant system for the internalization of substrates, including various cargo receptors and downstream interactors^8^. Such a mechanism is of great importance for delicate organs like the brain, where proper waste removal is a critical requirement to maintain an appropriate physiological function.

In summary, we here provide in vivo evidence that Dab2 is a major player during the removal of cargo generated by the brain parenchyma and therefore represents an important adaptor molecule mediating the clathrin-mediated internalization in BLECs.

## Methods

### Zebrafish strains

Experimental protocols and research on animals were designed and conducted according to the guidelines of the animal ethics committee of the University of Münster, Germany and approved by the responsible authorities (LANUV, North Rhine-Westphalia and veterinary office, Münster city). All experiments were performed in accordance with the ARRIVE guidelines. Zebrafish strains were maintained under standard husbandry conditions according to FELASA guidelines^31^. The following wild type and transgenic lines have been used in this study: *Casper*^32^, *Tg(lyve1:dsRed2)*^*nz101*33^, *Tg(flt4:mCitrine)*^*hu7135*34^, *Tg(flt1enh:tdTomato)*^*hu5333*35^, *Tg(fli1a:nEGFP)y7* ^36^, *Tg(mrc1a:mCitrine)*^mu409^.

### In situ hybridization, Immunofluorescent staining

The previously described *dab2* antisense mRNA probe is directed against a 1 kb fragment of the C-terminal end of the *dab2* cDNA^37^. For *dab2* detection, 100ng of the probe were hybridized using Casper embryos and following previously published protocols^38^. The optional antibody staining against *mCitrine* was performed after completion of the ISH protocol. Embryos were washed several times in 0,1% PBS-Triton 100X, followed by a blocking step for 1 h and an incubation with the primary antibody (anti-GFP, 1:200, Abcam, ab13970) in blocking buffer over night at room temperature. Samples were washed with 0,1% PBS-Triton 100X and afterwards incubated with the secondary antibody (anti-chicken-488, 1:200, Thermo scientific, A78948) in blocking buffer for 4 h at room temperature.

### Genome editing

The generation of the *dab2*^*mu410*^ deletion allele was performed by utilizing CRISPR/Cas9 mediated mutagenesis employing two guide RNAs (gRNA). The gRNA targeting the genomic region upstream of the transcriptional start site (5´-GGGCAGAAATGGGGTTATAA-3´) was designed with the chopchop algorithm (https://chopchop.cbu.uib.no/), and the gRNA targeting exon 3 (5´-GATGATGTTCAAGATGCAAG-3´) was chosen using the IDT gRNA design tool (https://eu.idtdna.com/site/order/designtool/index/CRISPR_SEQUENCE*).* For the mutagenesis, 71pg of each gRNA (annealed with tracr RNA) was injected together with 625pg of Cas9 protein (IDT) into the yolk of one-cell stage embryos with a fluorescent *flt4:mCitrine*;*flt1:tdTomato* background. The mutagenesis resulted in the deletion of 4.6 kb genomic fragment, spanning the putative promoter region of *dab2*.

### Genotyping

DNA extraction for genotyping was performed by lysing individual embryos in extraction buffer (50mM KCl, 2.5mM MgCl2, 10mM Tris pH 8.3, 0.45% v/v IGEPAL^®^ CA-630 (Sigma, #I8896), 0.45% v/v TWEEN^®^ 20, 0.01% Gelatin) containing 0.1 mg/ml proteinase K and incubation of the samples at 60°C for 1 hour and at 95°C for 15 minutes. For dye injections, embryos were genotyped before experiments, by extraction of DNA from clipped caudal fin tissue at 3dpf. Genotyping was done by touch-down PCR using three primers: a common reverse primer (5’-CTTGAACTGAAAGAGGGGTGGT-3’), a forward primer that only binds the wild type allele (5’-ACTGTTACTGTGGTACCAGCAG-3’), and a forward primer whose product is only amplified in the mutant allele (5’-CTTCCTCTTCACCCACTCTGAC-3’). Amplicons were visualized by gel electrophoresis.

### RNA-sequencing analysis

For analysis of *dab2* mRNA expression in BLECs, publicly available data (GSE97650) by Bower *et al.*^10^ was used. The heatmap of selected genes, clustered by Euclidean distance on their YuGene values, was created in R Studio (Version 4.1.3)^39^ utilizing the Complex Heatmap package (version 2.10.0) by Bioconductor 3.14 (https://bioconductor.org/packages/release/bioc/html/ComplexHeatmap.html*).*

### Quantification of ventrally sprouting ECs and of BLECs

Embryos were produced on the same day and kept under identical conditions until use. For the quantification of ventrally sprouting ECs, zygotic and maternal mutant embryos were imaged at 25-26hpf and subsequently, sprouts were quantified. The experiment was performed twice. Ventrally sprouting ECs are all sprouts that project towards the ventral side of the anterior-posterior axis (cf. Kim et al., 2012). Images were analysed in a randomized manner. To count the number of BLECs in siblings and mutant embryos, the embryos were imaged at 5dpf, and the number of *fli1a:nEGFP*-positive nuclei in *lyve1:dsRed* labelled BLECs was quantified. The results of three replicates were normalized to the numbers of the respective sibling embryos.

### Injection regimes

Injections were carried out using a pneumatic PicoPump with glass capillary needles (Science Products Gmbh, #GB100TF-10) generated by a Micropipette Puller (Shutter Instruments, #P-1000). Embryos were anaesthetized with 0.0168% tricaine and embedded (dorsal side up) in 1.5% low melting point agarose (ThermoFischer, #16520100), dissolved in embryo medium containing 0.0168% tricaine. Each embryo was injected into the cerebrospinal fluid with a total volume of 1nl per bolus. The needle was inserted at an angle and with care to not penetrate deeply into the parenchyma.

The following fluorescently-labelled macromolecules and concentrations were used: Alexa-Fluor-647 Cross-Adsorbed Secondary Antibody (2 mg/ml, ThermoFisher, #A21447); HiLyte Fluor 647-labeled Amyloid-β (1–40) (2 mg/ml, Anaspec, AS-60493), pHrodo Red Avidin (2 mg/ml, Thermo- Fisher, #P35362), pHrodo Red Dextran (2 mg/ml, ThermoFisher, P10361), Transferrin (2 mg/ml, ThermoFisher, T23366).

### Microscopy and image processing

Whole-mount in situ hybridization images were taken with a Nikon ECLIPSE Ni microscope. Embryos were transferred from PBT to 50% PBT/50% glycerol and 100% glycerol and placed on a glass slide.

Imaging for the description of the *dab2* phenotype, ventrally sprouting ECs quantification, and macromolecule internalization analysis was performed on a Leica SP8 confocal microscope employing Leica LAS X 3.5.6.21594 software and using 10x dry, 20x dry or 40x water immersion objectives. Embryos were anaesthetized with 0.0168% tricaine and embedded in 0.7% low melting point agarose (ThermoFischer, #16520100) containing 0.0168% tricaine. To inhibit pigment formation, embryos were treated with 1-phenyl-2-thiourea (PTU, Sigma Aldrich, #P7629) at < 24hpf. Confocal Z-stacks were acquired at 3 μm to 5 μm increments and processed using Fiji-ImageJ version 1.52 f.

Figures were assembled using Adobe Photoshop and Adobe Illustrator. All data were processed using raw images with brightness, colour and contrast adjusted for printing.

### Dye intensity quantification

All images from an imaging session were taken with the same parameters to allow direct comparison for quantification. The following macro was developed to quantify dye intensity with Fiji-ImageJ:

image = getTitle();

run(“Split Channels”);

selectWindow(“C3-“+image);

C3 = getTitle();

selectWindow(“C2-“+image);

C2 = getTitle();

selectWindow(“C1-“+image);

C1 = getTitle();

run(“Gaussian Blur…”, “sigma = 2 stack”);

setAutoThreshold(“Otsu dark”);

setOption(“BlackBackground”, true);

run(“Convert to Mask”, “method = Otsu background = Dark calculate black”);

run(“Close-“, “stack”);

run(“Dilate”, “stack”);

run(“Connected Components Labeling”, “connectivity = 8 type=[16 bits]”);

run(“Label Edition”);

selectWindow(“Label Edition”);

run(“glasbey_on_dark”);

setTool(“freeline”);

waitForUser(“Wait”);

run(“Close”);

run(“Label Size Filtering”, “operation = Greater_Than size = 1000”);

LabelImage = getTitle();

roiManager(“reset”);

run(“3D Manager”);

Ext.Manager3D_AddImage();

waitForUser(“Wait”);

selectWindow(C3);

roiManager(“Measure”);

selectWindow(C2);

roiManager(“Measure”);

roiManager(“reset”);

selectWindow(LabelImage);

waitForUser(“Wait”);

selectWindow(C3);

roiManager(“Measure”);

selectWindow(C2);

roiManager(“Measure”);

roiManager(“reset”);

### Statistical analysis

Statistical analysis was performed using R Studio (Version 4.1.3)^39^. For graphs, GraphPad Prism 9.3.1 was used. All data sets were tested for normality (Shapiro–Wilk) and equal variance. The p-values of normally distributed data with equal variance were determined with ANOVA and t test with Bonferroni correction for multiple comparisons. For data sets not following a normal distribution, p-values were determined using Kruskal-Wallis and Mann–Whitney test with Bonferroni correction for multiple comparisons. P values > 0.05 were considered not significant. * *p* ≤ 0.05, ** *p* ≤ 0.01, ***p ≤ 0.001, **** *p* ≤ 0.0001.

## Electronic supplementary material

Below is the link to the electronic supplementary material.

Supplementary Material 1

## Data Availability

The datasets generated during and/or analysed during the current study are available from the corresponding author on reasonable request. RNA-seq data, analysed during the current study and produced by Bower et al.^10^ are publicly available at GEO accession code GSE97650.
