# Supplementary material for: dab2 is required for the scavenging function of lymphatic endothelial cells in the zebrafish meninges
Source: Sci Rep. 2024 Nov 14;14:27942. doi: 10.1038/s41598-024-76590-9 (PMC11561233; doi:10.1038/s41598-024-76590-9)
Supplement: Supplementary file 1 — Supplementary Material 1 [file 41598_2024_76590_MOESM1_ESM.docx]

**Supplementary information**


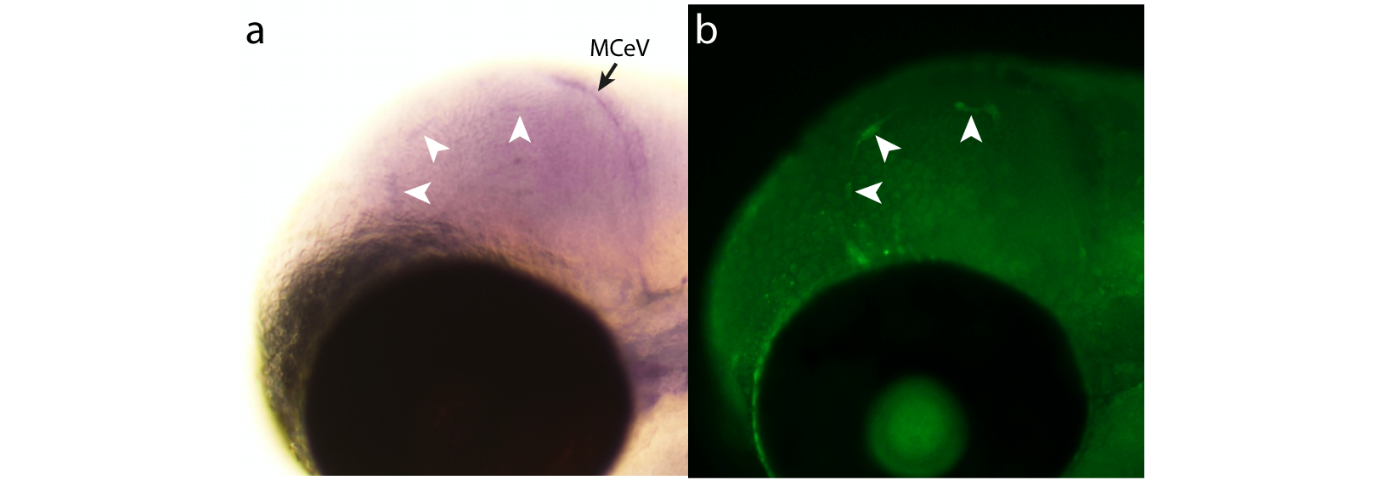


**Supplementary Figure 1: Cells positive for the *mrc1a* reporter are also positive for *dab2* expression**. **(a)** Bright field image and **(b)** fluorescent image of the same embryo that was stained by an ISH against *dab2* (a) and IF against *mCitrine* (b) in fixed *mrc1a:mCitrine* transgenic embryos at 86hpf. Note that all *mCitrine*-positive BLECs show expression of *dab2* transcripts. ISH: *in-situ* hybridization; IF: immunofluorescence; MCeV: middle cerebral vein


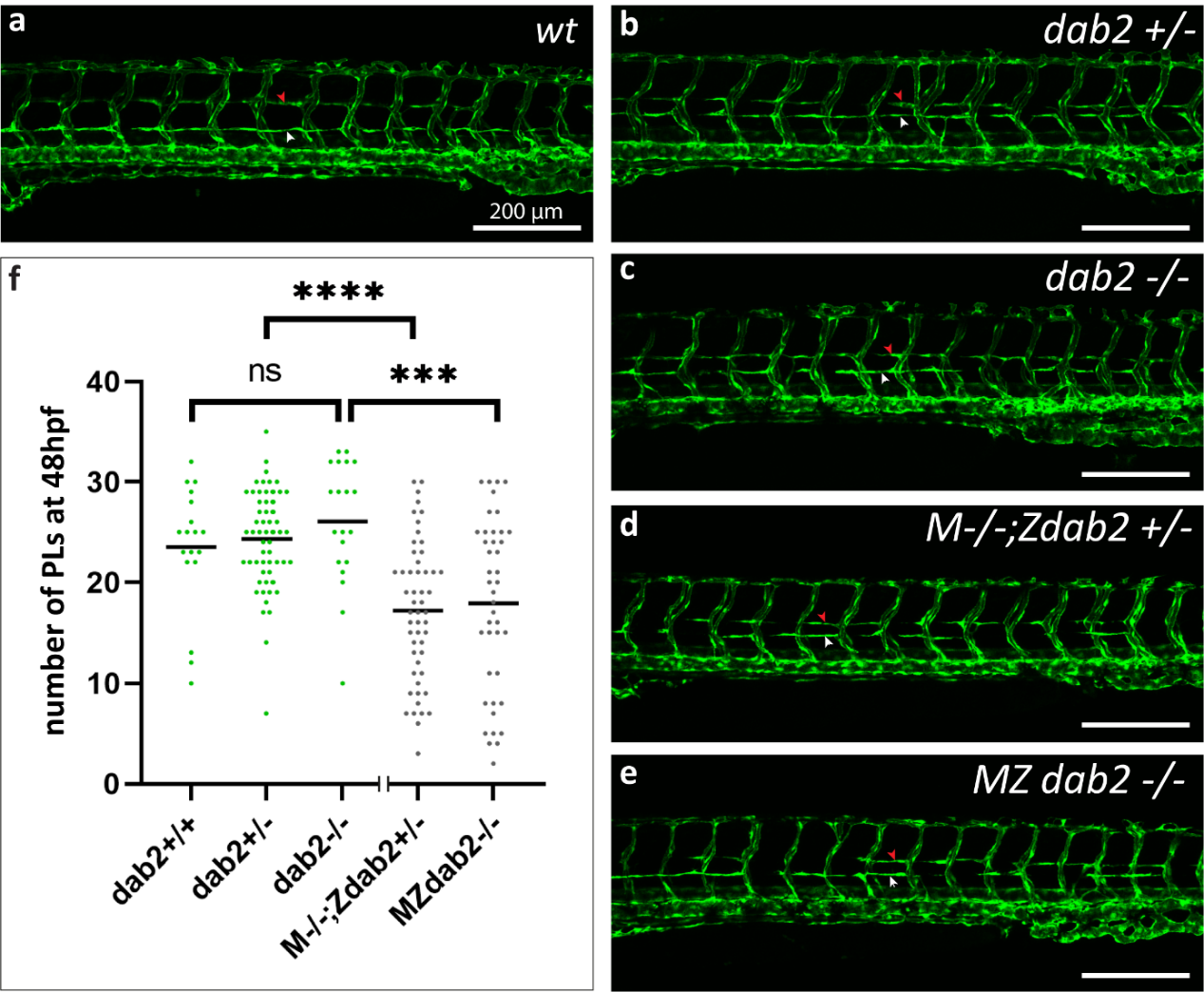


**Supplementary Figure 2: The number of PLs is reduced in embryos lacking maternally provided *dab2* mRNA.** **(a-e)** Maximum projection of the trunk of *flt4:mCitrine* positive embryos at 48hpf in lateral view, anterior to the left. White and red arrows highlight PLs on either body site. Wild type embryos (a), zygotic heterozygous (b), and zygotic mutant (c) embryos do not show overt differences with regard to PL formation at the horizontal myoseptum. Loss of maternal *dab2* mRNA in both zygotic heterozygous (M*-/-*;Z*dab2+/-* ) (d) and zygotic mutant (MZ*dab2-/-*) embryos (e) results in reduced PL numbers. **(f)** Quantification of the number of PLs in the whole trunk at 48hpf indicating significant differences in PL numbers upon loss of the maternal contribution of *dab2* mRNA (*dab+/+* median=25 (n=18) vs. *dab2-/-* median=27 (n=20) p-value = 0,2515; *dab2+/-* median=25 (n=57) vs. M*-/-*;Z*dab2+/-* median = 17 (n=53) p-value <0,0001; *dab2-/-* median=27 (n=20) vs MZ*dab2-/-* median=19 (n=42) p-value =0,0003 ; Mann-Whitney test. dpf, days post fertilization; PL, parachordal lymphangioblast.
